# Supplementary material for: Patterns of Intron Gain and Loss in Fungi
Source: PLoS Biol. 2004 Nov 30;2(12):e422. doi: 10.1371/journal.pbio.0020422 (PMC532390; doi:10.1371/journal.pbio.0020422)
Supplement: Table S1 — Also available at http://genes.mit.edu/NielsenEtAl/. (4.3 MB ZIP). [file pbio.0020422.st001.zip › NielsenEtAl/html/985.html]

AN6735.1.NCU06354.1.MG01450.1.FG02746.1


```
 CLUSTAL W (1.82) Multiple Sequence Alignments - Introns Inserted


Sequence 1: NCU06354.1	96 aa
Sequence 2: FG02746.1	96 aa
Sequence 3: MG01450.1	126 aa
Sequence 4: AN6735.1	91 aa
Alignment Length: 126 aa
Number Identitical Residues: 49 aa
Alignment Score (without introns) 2239


MG01450.1 	MTGRGGGGGRKILLPPI~NCIFKLLQS~HATVSIWLYEQLGIRIEGKIR0GFDEFMNLV~
NCU06354.1	MTGRGGGGNRRVLLPPI~NFLFRLLQQ~HSTVQIWLYEQLHIRIIGTIR0GFDEFMNLV0
FG02746.1 	MTGRGGGGGRRVLVPPI2NFIFKLLQS0HATVSVWLYEQLSIRIEGKIR0GFDEFMNLV~
AN6735.1  	MTGRGGGAARKTLLAPI~HFIFKLLQQ~RSTVSIWLYEQLAFRIEGKIR0GFDEFMNLV~
          	*******. *: *:.** : :*:***. ::**.:****** :** *.** ********* 

MG01450.1 	IDDAIEVKQITKDNTEEKRRPLA1YNRGDCEQQAEEGRTEGQGPHGPSMAAGLGMAHYDW
NCU06354.1	IDNAIEVKLVSKTNETEERRELG0-------QILLKG-------DNVSLIQSYSG-----
FG02746.1 	IDDAVEVKQITKTNDKESRRPLG1-------QILLKG-------DNVSLIQSAAS-----
AN6735.1  	IDDAVEVRLATKS-EEEKRRPLG~-----------MG---------ISIATTFLAQY---
          	**:*:**:  :*    *.** *.             *          *:           

MG01450.1 	GYGRPAPAGPA
NCU06354.1	-----------
FG02746.1 	-----------
AN6735.1  	-----------
          	
```
